# Spicy food intake and overweight/obesity in rural Southwest China: findings from a cross-sectional study

**DOI:** 10.3389/fnut.2025.1526775

**Published:** 2025-02-03

**Authors:** Huali Xiong, Peng Zhao, Fengxun Ma, Dayi Tang, Daiqiang Liu

**Affiliations:** ^1^Center for Mental Health of Rongchang District, Chongqing, China; ^2^Department of Public Health, Health Commission of Rongchang District, Chongqing, China; ^3^Department of Hepatobiliary Surgery, The People’s Hospital of Rongchang District, Chongqing, China; ^4^Department of Public Health, The People’s Hospital of Rongchang District, Chongqing, China; ^5^First Clinical College, Mudanjiang Medical College, Mudanjiang, Heilongjiang, China; ^6^Department of Hospital Information, The People’s Hospital of Rongchang District, Chongqing, China

**Keywords:** spicy food, intake, overweight, obesity, gender difference

## Abstract

**Background:**

Spicy food is an essential part of the culinary culture in rural southwest China, while little is known about the association between spicy food intake and overweight/obesity. This study was undertaken to explore the association between spicy food intake and overweight/obesity by a cross-sectional study.

**Method:**

We enrolled 2,995 individuals aged 30–79 years living in Rongchang, Chongqing municipality, southwest China from The China Multi-Ethnic Cohort Study, which was deemed to be the largest cohort study in southwest China. A multivariable-adjusted logistic regression model was applied to estimate the association between spicy food intake and overweight/obesity. Additionally, subgroup and sensitivity analyses were performed to verify the stability of the results.

**Results:**

A total of 81.67% of participants had the habit of consuming spicy food. The overall prevalence of overweight/obesity was 57.53%. Spicy food intake (*OR* = 2.913, 95%*CI*: 1.319–6.434) and frequency of spicy food intake (*OR* = 1.300, 95%*CI*: 1.164–1.452) were positively associated with overweight/obesity. Compared with the participants who never consumed spicy food, after adjusting for confounding factors, the adjusted *OR*s (95% *CI*s) in frequency of spicy food intake in 1–2 days/week, 3–5 days/week, 6–7 days/week subgroups were 3.985 (1.855–8.560), 4.381 (2.011–9.543), 6.515 (3.101–13.686), respectively. Subgroup analyses stratified by gender and age group, as well as sensitivity analyses, have consistently revealed a similar association between spicy food intake/the frequency of spicy food intake and overweight/obesity.

**Conclusion:**

This study could provide additional evidence for overweight and the obesity epidemic among rural adults in southwest China. Reducing spicy food intake might benefit from weight management.

## 1 Introduction

Unhealthy diet patterns have been associated with an increased risk of non-communicable metabolic diseases (1–3). Despite ongoing efforts by the Chinese government to prevent the overweight and obesity, the prevalence of obesity continues to increase among the Chinese population, especially in rural areas (4, 5). Excessive weight has been associated with increased mortality, cardiovascular diseases, higher blood pressure and blood sugar, musculoskeletal disorder, cancers (6–9) and disability-adjusted life-years attributable to excessive weight have more than doubled since 1990 (10). Healthy diets, physical activity, eHealth interventions, energy intake monitoring and other approaches are recommended for weight management (11, 12). Among them, dietary control is commonly used for weight reduction. It is worth noting that reducing the intake of certain foods is essential to combating obesity.

Spicy food such as chili sauce, chili oil, dried chili pepper, fresh chili pepper and other spices that increase spiciness are characterized by chemical irritants like capsaicin, which cause a burning, stinging, or tingling sensation (13). In the Asian diet, spicy food are often considered fundamental materials for enhancing flavor and taste (14), especially in India and China (14, 15). More than 30% of Chinese adults consume spicy foods, including chili peppers (16) every day. Numerous studies over the last few decades have examined the health benefits of spicy foods and their bioactive ingredients. Research has shown that spicy food consumption reduces the risk of cardiovascular disease, ischemic heart disease, cerebrovascular disease, all-cause mortality, and diabetes (16–19) and improved cognitive function in people with Alzheimer’s disease (20). On the other hand, higher consumption of spicy food may increase the likelihood of cancers, fractures and hyperuricemia (21–23).

There is a growing public interest in exploring whether spicy ingredients can help manage obesity due to the global obesity epidemic. However, the relationship between consuming spicy food and overweight/obesity remains controversial (5, 7, 24–26). Yang et al. (5) reported that the spicy food intake was associated with an increased risk of abdominal obesity, with fat energy intake playing a mediating role in this association. Xu et al. (24) also observed that the spicy food intake could elevate the risk of overweight and obesity in the population of Harbin. In contrast, the China Health and Nutrition Survey (25) indicated an inverse relationship between chili consumption and the prevalence of overweight or obesity among Chinese adults. Also, the association between spicy food intake and overweight/obesity in rural areas has not been fully explored. The lack of recent data in China necessitates further research in this field. Therefore, the current study was designed to explore the association between spicy food intake and overweight/obesity in rural southwest China.

## 2 Methods

### 2.1 Study design

The data were gathered from the baseline survey of The China Multi-Ethnic Cohort Study (27), which was the largest cohort study conducted by Sichuan university in southwest China from September 2018 to January 2019. Briefly in Rongchang region, participants were enrolled using a three-stage stratified random sampling method. First, four streets named Changyuan, Changzhou, Anfu, and Guangshun were randomly selected from 21 streets or towns; Second, 10 villages were randomly selected from each street; and third, 50 to 80 individuals were randomly selected ensuring the age and sex distribution accompanied with the age and sex distribution of Rongchang population. Ethic approvals from the ethics committee of Sichuan University (No. K2016038) were obtained, and all individuals signed informed consent forms before participating in the survey.

### 2.2 Study population

The study participants were recruited in the current study based on the following inclusion criteria: (1) aged between 30 to 79 years at the time of the investigation; (2) residents of Rongchang for over 6 months; (3) Han Nationality; (4) voluntarily participated in the survey and signed informed consent, agreed to provide biological samples and complete the follow up survey; (5) No mental illness and cognitive disorders, and absence of impaired expression ability. The individuals were excluded if they met the following criteria: missing data on general characteristics, questionnaires, physical examination, blood biochemical tests or spicy food intake. Overall, a total of 3,002 individuals were recruited in the baseline survey, but participants with missing data on dietary patterns were excluded. Ultimately, the study enrolled 2,995 participants for analysis to explore the associations between spicy food intake and overweight/obesity.

## 3 Data collection

All investigators, including face-to-face interviewers and physicians, underwent thorough training and passed an assessment by the quality control group prior to the survey’s commencement. Training included the use of tablets or computers for questionnaire surveys, verification and uploading of questionnaires, standardized methods for physical examinations, and procedures for sample collection and transportation. Verified questionnaires were uploaded after review by specialized staff. Trained post-graduates from Sichuan University reviewed 1% of the questionnaires daily, and any issues were immediately reported to the investigator for further review. All blood samples were analyzed by a third-party laboratory, Chongqing Dian Medical Testing Center Co., Ltd.

### 3.1 Assessment of covariates

General characteristics (age, gender, marital status, education level, job condition, average yearly income, etc.), life characteristics (smoking status, drinking status, physical activity, dietary information, etc.), and physical examination data (systolic blood pressure, SBP and diastolic blood pressure, DBP; height, body weight, etc.) were collected by well-trained interviewers. Venous blood samples were collected to measure the fasting blood glucose, lipid profiles, and other relevant biomarkers.

Age was expressed as M_50_[P_25_, P_75_]. Gender was categorized as “males” and “females.” Marital status was categorized as “married/cohabiting,” “separated/divorced/widowed/unmarried.” Education level was categorized as “primary school or below,” “junior middle school,” “high school or above.” Job condition was categorized as “farmers,” “others.” Average yearly income was categorized as “<20,000 yuan,” “20,000–59,999 yuan,” “60,000–99,999 yuan,” “≥100,000 yuan.” Smoking status was categorized as “never,” “current.” Drinking status was categorized as “never,” “current.” Hypertension (28) was defined as having an average SBP or DBP of ≥140 mmHg or ≥90 mmHg, respectively, based on three consecutive blood pressure measurement taken at 5 min intervals in a resting state, or having been diagnosed with hypertension by physicians or taking measures to lower their blood pressure (e.g., medications and exercises). Diabetes mellitus (29) was diagnosed when FPG levels over 7.00 mmol/L or when the individual had been diagnosed with diabetes mellitus by physicians or taking measures to lower their FPG level (e.g., medications, exercises). Dyslipidemia (30) was defined if any of the following four abnormalities were present: (1) total cholesterol ≥6.2 mmol/L; (2) triacylglycerol ≥2.3 mmol/L; (3) low density lipoprotein cholesterol ≥4.1 mmol/L; (4) high-density lipoprotein cholesterol <1.0 mmol/L. Metabolic equivalent tasks (METs) (31) were computed to estimate individuals’ physical activity level spent on working, transportation, household duties, and leisure time activities. All participants reported their intake of 13 main food groups by Food Frequency Questionnaire (FFQ) (32) in the baseline survey. Rice, wheat products, coarse grains, tubers, red meals (including processed), poultry, fish/sea food, eggs, fresh vegetables, soybean products, preserved vegetables, fresh fruits, dairy products were included in the 13 main food groups. For example, the detailed consumption of rice was measured through the following questions: “Did have rice in the past year” with two answer potions of “Yes” or “No.” Those who chose “Yes” and participants were further asked “During the past year, how often did you have rice?” and “What was the mass of each serving?.” The participants would report their frequency of rice consumption of “___times/day,” “___times/week,” “___times/month,” “___times/year” and “___g/serving.” Based on the Chinese Food Composition Table 2004, we calculated the average daily total energy intake for each individual (including protein, fat, and carbohydrate energy intake). A modified DASH diet score was calculated following the method created by Chiu et al. (33) and Chen et al. (34) with slight adaptions according to the CEMC data (32). The modified DASH diet focused on seven food groups, including the whole grains, fresh fruits, fresh vegetables, beans, dairy, red meat products and sodium. A score of 1–5 was assigned according to the quintile of the average food consumption. A score of 5 was assigned to the whole grains, fresh fruits, fresh vegetables, beans, dairy for the highest quintile, while red meat products and sodium were given a score of 5 for the lowest quintile. Then, the sum of seven food scores were represented an overall DASH score for the participants (35).

### 3.2 Assessment of spicy food intake

Similar to the Chinese Kadoorie Biobank study (36) and the CMEC study (34, 37), spicy food intake was defined as the consumption of any “spicy” condiment during cooking or eating, including “chili sauce,” “chili oil,” “fresh red peppers,” “dried chili peppers” or “other hot spices (curry or spicy spices, etc.)”. Individuals were enquired “How often have you eaten spicy food in the past month?” with the following five option: “never,” “<1 day/week,” “1–2 days/week,” “3–5 days/week” or “6–7 days/week.” Nobody chose the frequency of “<1 day/week.” Excluding those who chose “never,” individuals who reported weekly spicy food intake were further asked “What spicy food do you usually eat?” including “chili sauce,” “chili oil,” “fresh red peppers,” “dried chili peppers” or “other hot spices (curry or spicy spices, etc.)” and “How old have you been developing the habit of eating spicy food since approximately?.” The duration for spicy food intake was calculated as age at survey minus age at spicy food intake.

### 3.3 Assessment of overweight and obesity

Body mass index (BMI) was calculated as body weight divided by height squared (kg/m^2^). BMI value ranging from 24 to 27.9 kg/m^2^ were defined as overweight and BMI ≥ 28 kg/m^2^ was defined as obesity based on Expert Consensus on Obesity Prevention and Treatment in China (38).

## 4 Statistical analysis

Continuous variables which conform to normal distribution were presented as Means ± standard deviation (S.D.), otherwise, continuous variables with a non-normal distribution were presented as median and quartile [M (P_25_, P_75_)]. Categorical variables were presented as percentages (%). The difference in variables between overweight/obesity or the frequency of spicy food intake were evaluated using chi-square and ANOVA tests. The association between spicy food intake or the frequency of spicy food intake and overweight/obesity was examined using multivariable-adjusted logistic regression models, *OR* (odds ratios) and 95%*CI* (confidence interval) were calculated. Subgroup analysis by different gender and age group were also conducted. Kendall’s tau-b correlation index was conducted to investigate the correlation between the frequency of spicy food intake and body weight. After excluding participants with self-reported peptic ulcer disease, coronary heart disease, stroke, cancer and diabetes, sensitivity analyses was conducted to explore the association between spicy food intake/frequency of spicy food intake and overweight/obesity. Statistical analyses were performed using SPSS version 26.0 (IBM Corp., Armonk, NY, United States) with a statistic significance level set at *p* < 0.05.

## 5 Results

### 5.1 General characteristics of participants

The general characteristics of participants according to the overweight/obesity are presented in Table 1. The medium age of 2,955 participants was 49.00 [42.00, 60.00] years and the medium value of BMI was 24.62 [22.59, 26.88] and the overall prevalence of overweight/obesity was 57.53%. The prevalence of overweight/obesity differed significantly across various characteristics, including age, gender, educational level, job conditions, smoking status, drinking status, hypertension, diabetes, dyslipidemia, physical activity level and DASH score (*P* < 0.05).

**Table 1:** Characteristics of the participants according to the overweight and obesity.

Variables	Total	Overweight/obesity		*Z*/*χ*^2^ value	*P*
		No	Yes		
No. participants	2,995	1,272 (42.47)	1,723 (57.53)		
Age (years, M[P_25_, P_75_])	49.00 [42.00,60.00]	48.00 [43.00,61.00]	49.00 [42.00,60.0]	−6.476	<0.001
Gender, *n*(%)				20.868	<0.001
Males	1,497 (49.98)	574 (38.34)	923 (61.66)		
Females	1,498 (50.02)	698 (46.60)	800 (53.40)		
Marital status, *n*(%)				0.015	0.902
Married/cohabiting	2,703 (90.25)	1,147 (42.43)	1,556 (57.57)		
Separated/divorced/widowed/unmarried	292 (9.75)	125 (42.81)	167 (57.19)		
Educational level, *n*(%)				12.350	<0.001
Primary school or below	723 (24.14)	282 (39.00)	441 (61.00)		
Junior middle school	839 (28.01)	329 (39.21)	510 (60.79)		
High school or above	1,433 (47.85)	661 (46.13)	772 (53.87)		
Job conditions, *n*(%)				26.881	<0.001
Farmers	979 (32.69)	350 (35.75)	629 (64.25)		
Others	2,016 (63.71)	922 (45.73)	1,094 (54.27)		
Average yearly income, yuan (%)				0.450	0.502
<20,000	911 (30.42)	398 (43.69)	513 (56.31)		
20,000–59,999	1,087 (36.29)	452 (41.58)	635 (58.42)		
60,000–99,999	523 (17.46)	227 (43.30)	296 (56.50)		
≥100,000	474 (15.83)	195 (41.14)	279 (58.86)		
Smoking status, *n* (%)				6.872	0.009
Never	2,401 (80.17)	1,048 (43.65)	1,353 (56.35)		
Current	594 (19.83)	224 (37.71)	370 (62.29)		
Drinking status, *n* (%)				4.017	0.045
Never	1,469 (49.05)	651 (44.32)	818 (55.68)		
Current	1,526 (50.95)	621 (40.69)	905 (59.31)		
Hypertension, *n*(%)				109.355	<0.001
No	1,800 (60.10)	903 (50.17)	897 (49.83)		
Yes	1,195 (39.10)	369 (30.88)	826 (69.12)		
Diabetes, *n*(%)				47.446	<0.001
No	2,676 (89.35)	1,194 (44.62)	1,482 (55.38)		
Yes	319 (10.65)	78 (24.45)	241 (75.55)		
Dyslipidemia, *n*(%)				117.745	<0.001
No	2,168 (72.39)	1,052 (48.52)	1,116 (51.48)		
Yes	827 (27.61)	220 (26.60)	607 (73.40)		
Physical activity (METs-h/day, M[P_25_, P_75_])	15.58 [5.15, 28.88]	16.32 [6.56, 30.98]	14.80 [4.21, 29.10]	−2.533	0.011
Total energy intake (kcal/day, M[P_25_,P_75_])	1776.76	1755.84	1794.48	−1.776	0.076
	[1393.61, 2236.75]	[1382.64, 2186.86]	[1407.45,2260.59]		
DASH score, M[P_25_, P_75_]	23.00 [19.00,26.00]	23.00 [19.00, 26.00]	22.00 [19.00,26.00]	−2.359	0.018

DASH, dietary approaches to stop hypertension.

### 5.2 General characteristics associated with spicy food intake

The general characteristics associated with spicy food intake according to the overweight/obesity are presented in Table 2. Among the 2,995 participants, 81.67% claimed to have the habit of eating spicy food, with a higher prevalence of overweight/obesity compared to those who never ate spicy food (*P*<0.05). The prevalence of overweight/obesity increased with higher frequencies of spicy food intake compared to those who never ate spicy food (*P* < 0.05). The participants who consumed spicy food, such as chili sauce, chili oil, dried chili pepper, fresh chili pepper and other hot spices also had a higher prevalence of overweight/obesity (*P* < 0.05). Furthermore, participants with overweight/obesity reported a longer duration of spicy food intake (*P* < 0.05).

**Table 2:** The general characteristics associated with spicy food intake according to the overweight/obesity.

Variables	Total	Overweight/obesity		*Z*/*χ*^2^ value	*P*
		No	Yes		
Spicy food intake, *n*(%)				233.204	<0.001
No	549 (18.33)	393 (71.58)	156 (28.42)		
Yes	2,446 (81.67)	879 (35.94)	1,567 (64.04)		
Frequency of Spicy food intake, *n*(%)				235.959	<0.001
Never	549 (18.33)	393 (71.58)	156 (28.42)		
1–2 d/week	431 (14.39)	195 (45.24)	236 (54.76)		
3–5 d/week	266 (8.88)	111 (41.73)	155 (58.27)		
6–7 d/week	1,749 (58.40)	573 (32.76)	1,176 (67.24)		
Chili sauce, *n*(%)				209.454	<0.001
No	599 (20.00)	411 (68.61)	188 (31.39)		
Yes	2,396 (80.00)	861 (35.93)	1,535 (64.07)		
Chili oil, *n*(%)				13.519	<0.001
No	1,910 (63.77)	859 (44.97)	1,051 (55.03)		
Yes	1,085 (36.23)	413 (38.06)	672 (61.94)		
Dried chili pepper, *n*(%)				57.839	<0.001
No	1,705 (56.93)	826 (48.45)	879 (51.55)		
Yes	1,290 (43.07)	446 (34.57)	844 (65.43)		
Fresh chili pepper, *n*(%)				134.616	<0.001
No	712 (23.77)	436 (61.24)	276 (38.76)		
Yes	2,283 (76.23)	836 (36.62)	1,447 (63.38)		
Other hot spices, *n*(%)				23.404	<0.001
No	2,652 (88.55)	1,168 (44.04)	1,484 (55.96)		
Yes	343 (11.45)	104 (30.32)	239 (69.68)		
Duration for spicy food intake (years, M[P_25_, P_75_])	40.00 [32.00, 50.00]	39.00 [30.00, 49.00]	41.00 [34.00, 52.00]	−6.318	<0.001

### 5.3 Association of spicy food intake with overweight/obesity

Association between the general characteristics associated with spicy food intake and overweight/obesity is presented in Table 3. In the crude model, spicy food intake was positively associated with overweight/obesity (*OR* = 4.491, 95%*CI*: 3.666–5.502). After adjusting for the general factors associated with spicy food intake, including frequency of spicy food intake, chili sauce, chili oil, dried chili pepper, fresh chili pepper, other hot spices and duration for spicy food intake, spicy food intake (*OR* = 2.510, 95%*CI*: 1.181–5.334), frequency of spicy food intake (*OR* = 1.275, 95%*CI*: 1.147–1.418), consumed other hot spices (*OR* = 1.472, 95%*CI*: 1.127–1.923) and duration for spicy food intake (*OR* = 1.015, 95%*CI*: 1.009–1.021) remained positively associated with overweight/obesity. After progressively controlling for potential confounding factors, including gender, educational level, job conditions, average yearly income, smoking status, alcohol status, hypertension, diabetes, dyslipidemia, physical activity, DASH score, compared with those who never consumed spicy food, spicy food intake (*OR* = 2.913, 95%*C*I: 1.319–6.434) and frequency of spicy food intake (*OR* = 1.300, 95%*CI*: 1.164–1.452) remained positively associated with overweight/obesity.

**Table 3:** Association of spicy food intake with overweight and obesity.

Variables	*β*	S.D.	Walds*χ*^2^	*OR*	95%*CI*	*P*
Model 1						
Spicy food intake	1.502	0.104	210.264	4.491	3.666–5.502	<0.001
Model 2						
Spicy food intake	0.920	0.385	5.722	2.510	1.181–5.334	0.017
Frequency of Spicy food intake	0.243	0.054	20.190	1.275	1.147–1.418	<0.001
Chili sauce	0.276	0.318	0.751	1.318	0.706–2.460	0.386
Chili oil	−0.220	0.092	5.647	0.803	0.670–0.962	0.017
Dried chili pepper	0.126	0.090	1.953	1.134	0.951–1.353	0.162
Fresh chili pepper	−0.343	0.192	3.198	0.710	0.488–1.033	0.074
Other hot spices	0.387	0.136	8.065	1.472	1.127–1.923	0.005
Duration for spicy food intake	0.015	0.003	24.533	1.015	1.009–1.021	0.001
Model 3						
Spicy food intake	1.069	0.404	6.990	2.913	1.319–6.434	0.008
Frequency of Spicy food intake	0.262	0.056	21.557	1.300	1.164–1.452	<0.001
Chili sauce	0.090	0.335	0.072	1.094	0.567–2.111	0.789
Chili oil	−0.178	0.097	3.382	0.837	0.693–1.012	0.066
Dried chili pepper	0.095	0.094	1.030	1.100	0.915–1.321	0.310
Fresh chili pepper	−0.335	0.199	2.833	0.715	0.484–1.057	0.092
Other hot spices	0.408	0.142	8.308	1.504	1.139–1.984	0.004
Duration for spicy food intake	0.000	0.004	0.002	1.000	0.993–1.007	0.967

OR, odds ration.

CI, confidence interval.

DASH, dietary approaches to stop hypertension.

REF, reference.

Model 1, crude model without adjustment.

Model 2, adjusted for Model 1 plus frequency of spicy food intake, chili sauce, chili oil, dried chili pepper, fresh chili pepper, other hot spices, duration for spicy food intake.

Model 3, adjusted for Model 2 plus gender, educational level, job conditions, average yearly income, smoking status, alcohol status, hypertension, diabetes, dyslipidemia, physical activity, DASH score (all the adjusted variables showed statistically significant difference in prevalence of overweight and obesity in Tables 1, 2).

### 5.4 The correlation between the spicy food intake and body weight

**Figure 1:**
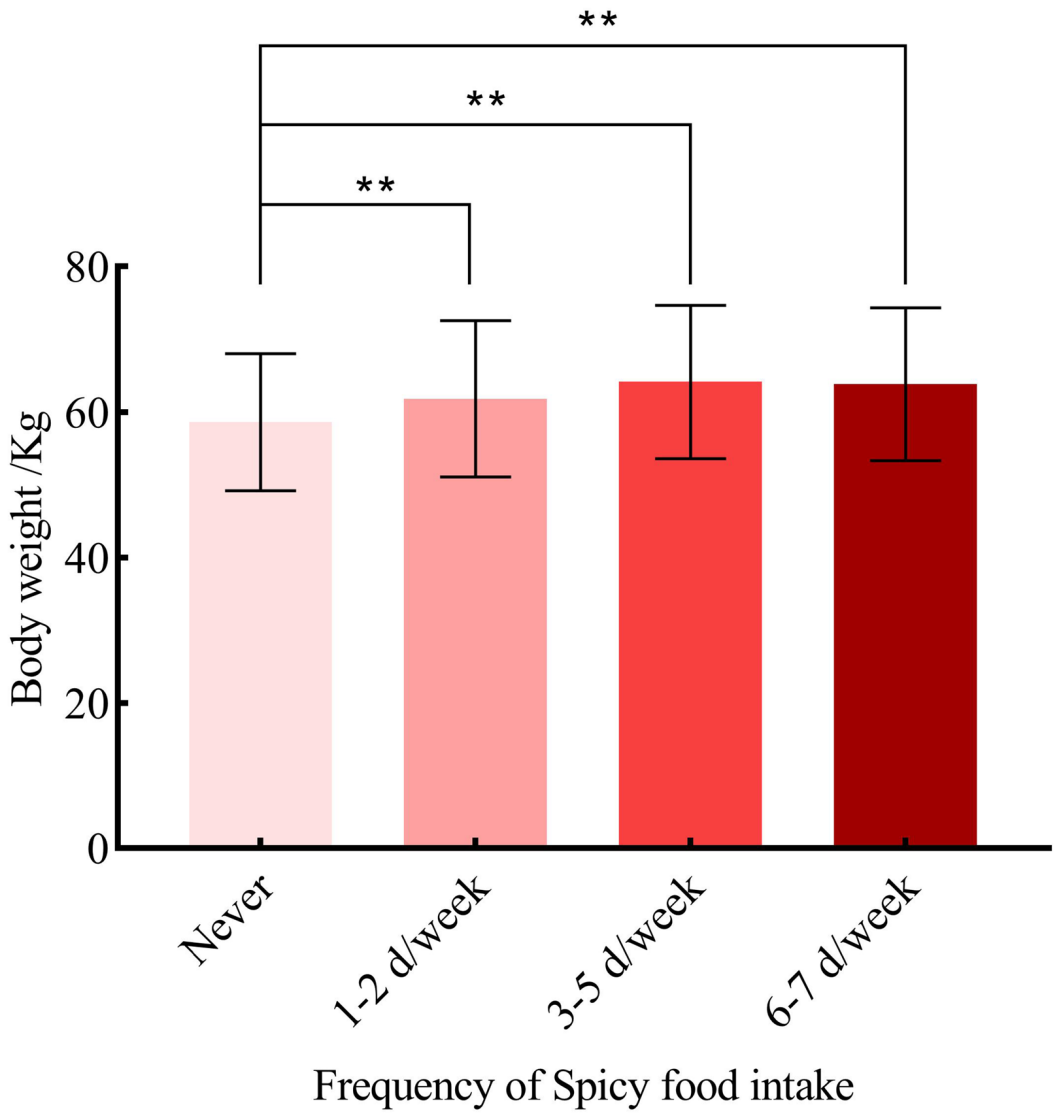
The correlation between frequency of spicy food intake and body weight.

Participants who consumed spicy food had a higher body weight with a medium of 62.60 [56.00, 70.00] kg, compared to those who never consumed spicy food with a medium body weight of 57.20 [51.50, 64.30] (*Z* = −10.326, *P*<0.001). Compared with the participants who consumed no spicy food, the body weight in 1–2 days/week (60.60 [54.30, 68.00]), 3–5 days/week (64.00 [56.65, 71.00]), 6–7 days/week (63.00 [56.50, 70.00]) subgroups of spicy food intake frequency were significantly different (*H* = 123.549, *P* < 0.001) (Figure 1). Kendall’s Tau-b correlation index also demonstrated a significant positive correlation between spicy food intake frequency and body weight (Kendall’s Tau-b value = 0.138, *p* < 0.001). The prevalence of overweight/obesity increased with the frequency of spicy food consumption, rising from 54.76% in the 1–2 days/week to 58.27% in the 3–5 days/week and 67.18% in the 6–7 days/week, compared to those who never consumed spicy food (28.42%) (*χ*^2^_trend_ = 235.039, *P* < 0.001).

### 5.5 Subgroup analysis

#### 5.5.1 Association between spicy food intake frequency and overweight and obesity

The general characteristics of participants according to the spicy food intake frequency are presented in Table 4. The difference among the characteristics including age, gender, educational level, average yearly income, smoking status, drinking status, hypertension, dyslipidemia, physical activity level, total energy intake, DASH score and BMI were significantly different between different spicy food intake frequency (*P* < 0.05).

**Table 4:** The general characteristics of participants according to the spicy food intake frequency.

Variables	Total	Frequency of spicy food intake				*H*/*χ*^2^ value	*P_trend_*
		Never	1–2 d/week	3–5 d/week	6–7 d/week		
No. participants	2,995	549	431	266	1749		
Age (years, M[P_25_, P_75_])	49.00 [42.00,60.00]	48.00 [43.00,61.00]	47.00 [40.00,55.00]	46.00 [38.00,55.00]	50.00 [43.00,61.00]	39.597	<0.001
Gender, *n*(%)							
Males	1,497 (49.98)	243 (44.26)	207 (48.03)	153 (57.52)	894 (51.11)	7.601	0.006
Females	1,498 (50.02)	306 (55.74)	224 (51.97)	113 (42.48)	855 (48.89)		
Marital status, *n*(%)							
Married/cohabiting	2,703 (90.25)	502 (91.44)	390 (90.49)	237 (89.10)	1,574 (89.99)	0.897	0.344
Separated/divorced/widowed/unmarried	292 (9.75)	47 (8.56)	41 (9.51)	29 (10.90)	175 (10.01)		
Educational level, *n*(%)							
Primary school or below	723 (24.14)	118 (21.49)	104 (24.13)	64 (24.06)	437 (24.99)	6.982	0.008
Junior middle school	839 (28.01)	158 (28.78)	102 (23.67)	45 (16.92)	534 (30.53)		
High school or above	1,433 (47.85)	273 (49.73)	225 (52.2)	157 (59.02)	778 (44.48)		
Job conditions, *n*(%)							
Farmers	979 (32.69)	171 (31.15)	139 (32.25)	78 (29.32)	591 (33.79)	1.044	0.307
Others	2016 (63.71)	378 (68.85)	292 (67.75)	188 (70.68)	1,158 (66.21)		
Average yearly income, yuan (%)							
<20,000	911 (30.42)	210 (38.25)	120 (27.84)	68 (25.56)	513 (29.33)	11.25	<0.001
20,000–59,999	1,087 (36.29)	187 (34.06)	156 (36.19)	106 (39.85)	638 (36.48)		
60,000–99,999	523 (17.46)	93 (16.94)	82 (19.03)	46 (17.29)	302 (17.27)		
≥100,000	474 (15.83)	59 (10.75)	73 (16.94)	46 (19.29)	296 (16.92)		
Smoking status, *n*(%)							
Never	2,401 (80.17)	495 (90.16)	356 (82.60)	199 (74.81)	1,351 (77.24)	42.35	<0.001
Current	594 (19.83)	54 (9.84)	75 (17.40)	67 (25.19)	398 (22.76)		
Drinking status, *n*(%)							
Never	1,469 (49.05)	329 (59.93)	204 (47.33)	120 (45.11)	816 (46.66)	21.733	<0.001
Current	1,526 (50.95)	220 (40.07)	227 (52.67)	146 (54.89)	933 (53.34)		
Hypertension, *n*(%)							
No	1800 (60.1)	354 (64.48)	281 (65.20)	167 (62.78)	998 (57.06)	14.518	<0.001
Yes	1,195 (39.1)	195 (35.52)	150 (34.80)	99 (37.22)	751 (42.94)		
Diabetes, *n*(%)							
No	2,676 (89.35)	498 (90.71)	396 (91.88)	233 (87.59)	1,549 (88.56)	3.772	0.052
Yes	319 (10.65)	51 (9.29)	35 (8.12)	33 (12.41)	200 (11.44)		
Dyslipidemia, *n*(%)							
No	2,168 (72.39)	430 (78.32)	307 (71.23)	183 (68.80)	1,248 (71.36)	7.024	0.008
Yes	827 (27.61)	119 (21.68)	124 (28.77)	83 (31.20)	501 (28.64)		
Physical activity (METs-h/day, M[P_25_, P_75_])	15.58 [5.15, 28.88]	14.88 [4.21, 27.83]	15.74 [6.34, 28.24]	19.17 [7.40, 32.15]	15.07 [4.71, 30.57]	8.272	0.041
Total energy intake (kcal/day, M[P_25_,P_75_])	1776.76 [1393.61, 2236.75]	1722.37 [1323.96, 2025.30]	1758.61 [1374.92, 2155.62]	1799.33 [1408.39, 2227.24]	1807.62 [1422.62, 2288.14]	12.792	0.005
DASH score, M[P_25_, P_75_]	23.00 [19.00,26.00]	23.00 [19.00, 26.00]	24.00 [20.00, 27.00]	24.00 [21.00, 27.00]	23.00 [19.00, 26.00]	44.944	<0.001
BMI (kg/m2, M [P_25_, P_75_])	24.62 [22.59,26.88]	23.02 [21.41, 24.61]	24.42 [22.31, 26.63]	24.64 [22.85, 26.65]	25.16 [23.21, 27.24]	185.759	<0.001

DASH, dietary approaches to stop hypertension.

BMI, body mass index.

The *OR*s of spicy food intake frequency and overweight/obesity is presented in Table 5. Compared with the participants who never consumed spicy food (reference group), the crude *OR*s (95%*CI*s) in 1–2 days/week, 3–5 days/week, 6–7 days/week subgroups of spicy food intake frequency were 3.049 (2.338–3.975), 3.518 (2.590–4.778), 5.170 (4.188–6.383), respectively. After progressively controlling for potential confounding factors, in the final model (model 4), the adjusted *OR*s (95%*CI*s) in 1–2 days/week, 3–5 days/week, 6–7 days/week subgroups were 3.985 (1.855–8.560), 4.381 (2.011–9.543), 6.515 (3.101–13.686), respectively.

**Table 5:** The ORs of spicy food intake frequency and overweight and obesity.

Frequency of spicy food intake	*β*	S.D.	Walds *χ*^2^	*OR*	95%*CI*	*P*
Model 1						
Never				REF.		
1–2 d/week	1.115	0.135	67.835	3.049	2.338–3.975	<0.001
3–5 d/week	1.258	0.156	64.803	3.518	2.590–4.778	<0.001
6–7 d/week	1.643	0.107	233.695	5.170	4.188–6.383	<0.001
Model 2						
Never				REF.		
1–2 d/week	1.196	0.370	10.433	3.307	1.601–6.834	0.001
3–5 d/week	1.306	0.377	12.017	3.692	1.764–7.727	0.001
6–7 d/week	1.661	0.359	21.388	5.263	2.604–10.638	<0.001
Model 3						
Never				REF.		
1–2 d/week	1.174	0.374	9.875	3.235	1.555–6.727	0.002
3–5 d/week	1.284	0.380	11.408	3.611	1.714–7.609	0.001
6–7 d/week	1.641	0.362	20.545	5.163	2.539–10.499	<0.001
Model 4						
Never				REF.		
1–2 d/week	1.383	0.390	12.561	3.985	1.855–8.560	<0.001
3–5 d/week	1.477	0.397	13.832	4.381	2.011–9.543	<0.001
6–7 d/week	1.874	0.379	24.485	6.515	3.101–13.686	<0.001

OR, odds ration.

CI, confidence interval.

REF, reference.

DASH, dietary approaches to stop hypertension.

Model 1, crude model without adjustment.

Model 2, adjusted for Model 1 plus chili sauce, chili oil, dried chili pepper, fresh chili pepper, other hot spices, duration for spicy food intake.

Model 3, adjusted for Model 2 plus age, gender, marital status, educational level, job conditions, average yearly income.

Model 4, adjusted for Model 3 plus smoking status, alcohol status, hypertension, diabetes, dyslipidemia, physical activity, total energy intake and DASH score.

#### 5.5.2 Association between spicy food intake frequency and overweight/obesity in different gender and age

**Figure 2:**
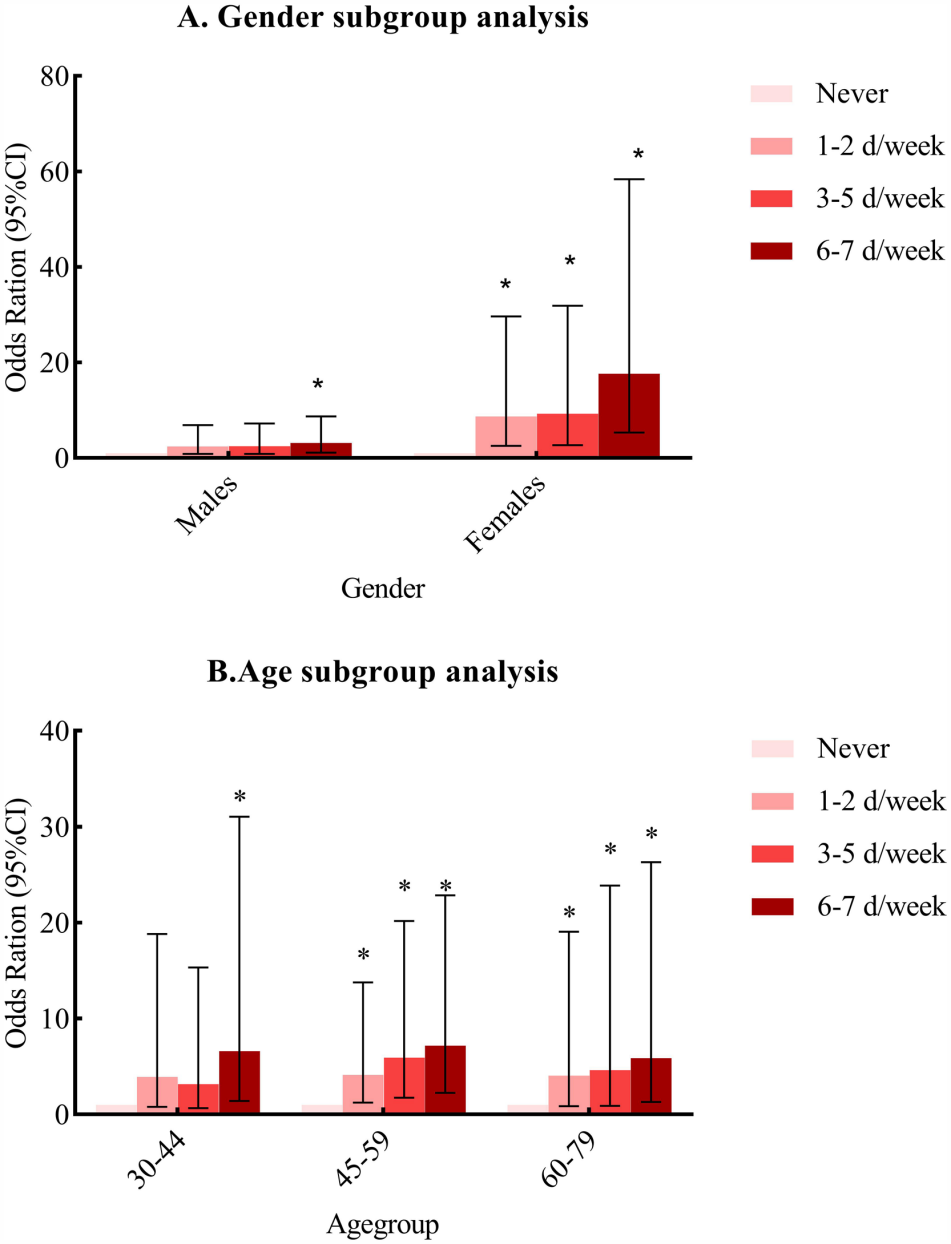
Subgroup analysis by **(A)** gender and **(B)** age. *Compared with those who never consumed spicy food, *p* < 0.05.

As shown in Figure 2, after adjusting for confounding factors, including chili sauce, chili oil, dried chili pepper, fresh chili pepper, other hot spices, duration for spicy food intake, age, gender, marital status, educational level, job conditions, average yearly income, smoking status, alcohol status, hypertension, diabetes, dyslipidemia, physical activity, total energy intake and DASH score, a similar association was found in males and females. However, statistically significant *OR*s (95%CIs) were observed only in males in the 6–7 days/week subgroup (*OR* = 3.148, 95%*CI*: 1.142–8.676, *p* = 0.027) and in females in the 1–2 days/week (*OR* = 8.680, 95%*CI*: 2.540–29.659, *P* < 0.001), 3–5 days/week (*OR* = 9.256, 95%*CI*: 2.686–31.888, *P* < 0.001), 6–7 days/week (*OR* = 17.634, 95%*CI*: 5.330–58.346, *P* < 0.001), (Figure 2A). Regarding age groups, a similar association was found across the 30–44 years, 45–59 years, and 60–79 years age groups, where a higher frequency of spicy food intake was associated with a greater risk of overweight/obesity, (Figure 2B).

### 5.6 Sensitivity analyses

After excluding participants with self-reported peptic ulcer disease, coronary heart disease, stroke, cancer and diabetes, a total of 2,447 participants were included in the sensitivity analyses. Compared with the individuals who never consumed spicy food, spicy food intake was positively associated with overweight/obesity (*OR* = 6.099, 95%*CI*: 2.771–13.421). The adjusted *OR*s (95% *CI*s) for frequency of spicy food intake in 1–2 days/week, 3–5 days/week, 6–7 days/week subgroups were 4.360 (1.922–9.980), 4.364 (2.024–10.635), 6.632 (3.005–14.635), respectively. Notably, the *OR* values in the sensitivity analysis are higher compared to previous analyses.

## 6 Discussion

The current study focused on the association between spicy food intake and overweight/obesity among rural Chinese adults in southwest China and found that spicy food intake and the frequency of spicy food intake were positively correlated with overweight/obesity, which was in contrast to previous studies from Western countries (14, 39–42). A total of 81.67% of participants had the habit of spicy food consumption, indicating that spicy food is quite popular in southwest China (37). The China Kadoorie Biobank study, one of the largest cohort studies in the world, demonstrated that covariate-adjusted BMI increased significantly with the frequency of spicy food consumption, the degree of pungency in spicy food, and the duration of spicy food consumption in both males and females (43). The Qingdao study (24) also found that spicy food intake could increase the risk of overweight and obesity, with females who preferred a meat dietary pattern and a higher frequency of spicy food intake having the highest risk of overweight and obesity. A study from the Rural Diabetes, Obesity and Lifestyle Study (7) conducted in Henan province identified a positive relationship between spicy flavor, spicy food intake frequency, and general obesity in a rural adult Chinese population.

Conversely, a study from the China Health and Nutrition Survey (25) which conducted a follow-up on 12,970 participants for over 20 years, demonstrated that chill consumption was inversely associated with the incidence of overweight/obesity, independent of overall dietary pattern, energy intake and lifestyle factors. Studies from Western countries also confirmed that the consumption of spicy food was potentially beneficial for weight management (14, 39–42). Chili peppers consumption might decrease energy intake, increase energy expenditure, and enhance fat oxidation (41, 42), in which to achieve the purpose of weight control. The inconsistent results of population studies may be related to the differences in study design, study participants, classification standards of spicy food intake and dietary habits. The impact of consuming spicy food on BMI weight management was not found in the current study, as participants consider consuming spicy food at different frequencies as a lifelong dietary habit.

There are no definitive mechanistic studies to prove that spicy food intake was associated with an increased the risk of developing overweight/obesity. Nevertheless, there were several potential mechanisms which could characterize the association between spicy food intake and overweight/obesity. Hedonically, spicy food are thought to increase the palatability of food by improving taste, flavor, color, odor and believed to resist the loss of appetite (14). There is a higher likelihood of meat-dominated dietary patterns compared to vegetables along with chili intake, and excessively consuming spicy, fatty meat food may increase the risk of overweight/obesity (44). Chili oil and/or sauce are commonly used as a sauce in Chinese cuisine, and the consumption of chili oil could simultaneously increase the consumption of carbohydrate rich food to relieve their burning sensation, potentially leading to weight gain (7, 44). Additionally, the consumption of hot pepper could increase cravings for desserts, which can reduce burning sensation on the tongue, while consumption of spicy foods can also trigger cravings for cold drinks. The extra intake of sweets and beverages in a pungent diet increases the risk of developing obesity. Furthermore, many studies have demonstrated that individuals with high consumption of spicy food were more preferred to be drinkers (45, 46). Previous studies had demonstrated that alcohol drinking is positively associated with obesity (47, 48). In conclusion, the excessive intake of fat, carbohydrates, oil, desserts and alcohol along with spicy food could increase the risk of overweight/obesity. After adjusting for total energy intake and DASH dietary pattern as confounding factors in the current study, the analogous relationship between spicy food intake and overweight/obesity remained.

The humid climate of Chongqing municipality has led residents to develop a long-standing habit of consuming spicy food. Depending on Traditional Chinese Medicine, consuming spicy food helps expel dampness (referred to as “shi qi” in Chinese) from the body. Examining the impact of spicy food intake on overweight/obesity in different regions and among individuals with varying dietary habits and culture has more practical guiding significance for weight management. Chongqing municipality is renowned worldwide for its hot pot with heavy salt and/or oil used for flavor or preservation, this might have contributed to the overweight/obesity of the Chongqing population. We continuously ponder why our small-scale study consistently yields results similar to those of the China Kadoorie Biobank study, while differing from findings in the China Health and Nutrition Survey. A key observation lies in the ubiquity of spicy food consumption throughout the daily meals of ordinary individuals. For instance, during breakfast, staples like noodles and spicy pickled vegetables prominently feature spiciness. At lunch, the use of chili sauce, chili oil, dried/fresh chili peppers, garlic, and ginger is widespread in stir-fry dishes, enhancing both color and flavor. In the evening, hot pot, a culinary favorite in Chongqing, takes center stage for dinner. Speaking emphatically, the consumption of spicy food is as pervasive in Chongqing as drinking water, whether the spiciness is mild or pronounced. The influence of spicy food consumption on the health of Chongqing residents extends well beyond issues of overweight and obesity. All the aforementioned insights are speculative, and the exact mechanism underlying the association between spicy food intake and overweight/obesity needs further study.

The present study offers novel insights into the risk factors associated with overweight/obesity in the rural southwest China, and it indeed exhibits several notable strengths. Notably, we are pioneers in analyzing the correlation between spicy food intake and overweight/obesity within the general population residing in regions with widespread spicy food consumption. Furthermore, we conducted subgroup and sensitivity analyses, rendering our exploration more representative. The insights derived from this study contribute to understanding the nuanced relationship between spicy food intake and overweight/obesity. Additionally, we maintain a high data quality by relying on experienced investigators during surveys. Moreover, our study benefits from the meticulous adjustment for various confounding factors, enhancing the robustness of the association between spicy food intake and overweight/obesity. However, it is imperative to acknowledge certain limitations in our research. Firstly, the cross-sectional nature of the survey prevents us from inferring causal relationships. The subsequent data collected through cohort study could be effectively utilized to elucidate the correlation between the consumption of spicy food and the progression of chronic diseases, including obesity. Secondly, although dietary patterns are recognized influence factors of overweight and obesity, we did not analyze the confounding effects of specific dietary patterns; instead, we utilized total energy intake. Thirdly, our study did not delve into the individual consumption of different spicy foods, and further research is essential to elucidate the specific mechanisms underlying capsaicin’s impact on overweight and obesity. Fourthly, the data regarding spicy food consumption were based on self-reported information from the participants, which could introduce reporting bias. Additionally, no comprehensive dietary epidemiological surveys or pertinent laboratory analyses were conducted. Lastly, our focus solely on Chongqing in southwest China raises concerns about the generalizability of our findings to the entire Chinese population. Future research across diverse regions is warranted to comprehensively explore the relationship between spicy food and overweight/obesity.

In this study, the criteria we applied for overweight (BMI: 24–27.9 kg/m^2^) and obesity (BMI ≥ 28 kg/m^2^), (38) were issued by National Health and Family Planning Commission of the People’s Republic of China (Now: National Health Commission of the People’s Republic of China) in 2013, which was different from the World Health Organization’s criteria for determining overweight (BMI: 25–29.99 kg/m^2^) and obesity (BMI ≥ 30 kg/m^2^). December 31, 2024, General Office of the National Health Commission of the People’s Republic of China released guidelines for weight management (2024 edition) (49), the guidelines clarified the standard for determining overweight (BMI: 24–27.9 kg/m^2^) and obesity (BMI ≥ 28 kg/m^2^) by BMI, which was consistent with the standards from 2013. Additionally, the guidelines have explicitly stated that dietary intervention is the primary means of weight management. Spicy food, as a common dietary habit in the southwest China and has been found to increase the risk of overweight and obesity. It remains to be elucidated whether the heightened risk of overweight and obesity is attributable to spicy food intake or the concomitant excessive energy intake that may stem from a preference for spicy food dietary habits. Nevertheless, curtailing spicy food intake could be pivotal in preventing the onset of overweight/obesity. Policymakers in regions with analogous culinary traditions should develop and implement multifaceted public health policies. These should encompass a spectrum of initiatives, such as bolstering educational efforts and public awareness campaigns on wholesome dietary practices, advocating for increased physical activity, conducting timely risk assessments and initiating early interventions, and instituting robust monitoring and early warning systems to address these health concerns effectively.

## 7 Conclusion

The current study demonstrated that spicy food intake and the frequency of spicy food intake are positively associated with overweight/obesity. The government should contemplate strategies for preventing the widespread occurrence of overweight and obesity by promoting healthy spicy food consumption. The correlation between spicy food intake and the incidence of overweight/obesity may be substantiated through future cohort studies. Policymakers should craft and implement policies aimed at mitigating the overweight and obesity epidemic, focusing on prevention, education, surveillance, and intervention strategies.

## Data Availability

The original contributions presented in the study are included in the article/Supplementary material, further inquiries can be directed to the corresponding author.
